# Choline-deficient, high-fat diet-induced MASH in Göttingen Minipigs: characterization and effects of a chow reversal period

**DOI:** 10.1152/ajpgi.00120.2024

**Published:** 2024-07-23

**Authors:** Henning Hvid, Sara T. Hjuler, Pierre Bedossa, Dina G. Tiniakos, Ioannis Kamzolas, Lea M. Harder, Yaxin Xue, James W. Perfield, Rikke K. Kirk, Markus Latta, Lars F. Mikkelsen, Henrik D. Pedersen, LITMUS Investigators

**Affiliations:** ^1^Research and Early Development, Novo Nordisk A/S, Maaloev, Denmark; ^2^LIVERPAT, Paris, France; ^3^Department of Pathology, Aretaieion Hospital, Medical School, National and Kapodistrian University of Athens, Athens, Greece; ^4^Translational and Clinical Research Institute, Faculty of Medical Sciences, Newcastle University, Newcastle upon Tyne, United Kingdom; ^5^Wellcome Trust-MRC Institute of Metabolic Science, University of Cambridge, Cambridge, United Kingdom; ^6^European Molecular Biology Laboratory, European Bioinformatics Institute, Wellcome Genome Campus, Hinxton, United Kingdom; ^7^Lilly Research Labs, Eli Lilly and Company, Indianapolis, Indiana, United States; ^8^Ellegard Göttingen Minipigs A/S, Dalmose, Denmark

**Keywords:** choline-deficient high-fat diet, Göttingen Minipigs, liver, MASH, MASLD

## Abstract

The prevalence of metabolic dysfunction-associated steatotic liver disease (MASLD) and metabolic dysfunction-associated steatohepatitis (MASH) is increasing, and translational animal models are needed to develop novel treatments for this disease. The physiology and metabolism of pigs have a relatively high resemblance to humans, and the present study aimed to characterize choline-deficient and high-fat diet (CDAHFD)-fed Göttingen Minipigs as a novel animal model of MASLD/MASH. Göttingen Minipigs were fed CDAHFD for up to 5 mo, and the phenotype was investigated by the analysis of plasma parameters and repeated collection of liver biopsies. Furthermore, changes in hepatic gene expression during the experiment were explored by RNA sequencing. For a subset of the minipigs, the diet was changed from CDAHFD back to chow to investigate whether the liver pathology was reversible. Göttingen Minipigs on CDAHFD gained body weight, and plasma levels of cholesterol, AST, ALT, ALP, and GGT were increased. CDAHFD-fed minipigs developed hepatic steatosis, inflammation, and fibrosis, which in 5 of 16 animals progressed to cirrhosis. During an 11-wk chow reversal period, steatosis regressed, while fibrosis persisted. Regarding inflammation, the findings were less clear, depending on the type of readout. MASH Human Proximity Scoring (combined evaluation of transcriptional, phenotypic, and histopathological parameters) showed that CDAHFD-fed Göttingen Minipigs resemble human MASLD/MASH better than most rodent models. In conclusion, CDAHFD-fed minipigs develop a MASH-like phenotype, which, in several aspects, resembles the changes observed in human patients with MASLD/MASH. Furthermore, repeated collection of liver biopsies allows detailed characterization of histopathological changes over time in individual animals.

**NEW & NOTEWORTHY** The physiology and metabolism of pigs have a relatively high resemblance to humans. This study characterizes a new animal model of MASLD/MASH using CDAHFD-fed Göttingen Minipigs. Göttingen Minipigs fed CDAHFD gained weight and developed hepatic steatosis, inflammation, fibrosis, and cirrhosis. After an 11-wk chow-reversal period, hepatic steatosis and some inflammatory parameters reversed. Combined evaluation of phenotypic, transcriptional, and histological parameters revealed the minipig model showed a higher resemblance to human disease than many rodent models.

## Graphical abstract

**Figure:**
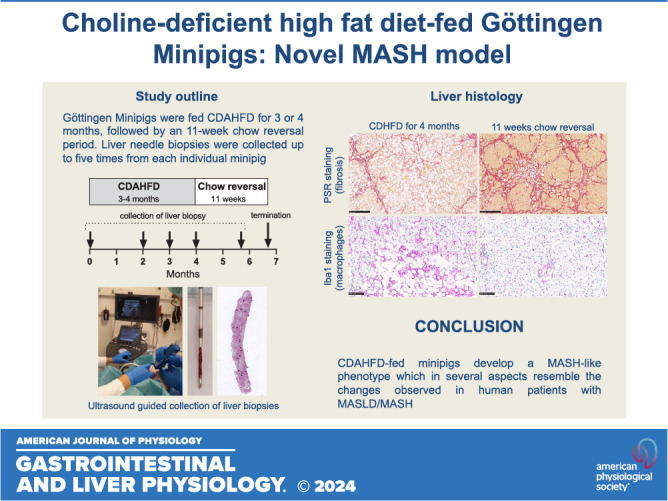

## INTRODUCTION

Metabolic dysfunction-associated steatotic liver disease (MASLD) is used as a general description for different stages of liver disease with distinct histological characteristics, ranging from simple nonalcoholic fatty liver and the more severe metabolic dysfunction-associated steatohepatitis (MASH), characterized by hepatic steatosis, inflammation, ballooning hepatocytes, and often hepatic fibrosis, to end-stage liver disease, characterized by cirrhosis and/or hepatocellular carcinoma (1, 2). It is estimated that ∼25% of the general population have MASLD and that 1.5–6.5% have MASH (3). These prevalences are concerning, as patients with MASLD have higher all-cause mortality (4), and MASH has become a common etiology for liver transplantation in the United States (5). There are currently no pharmacological treatments available, but multiple biotech- and pharmaceutical companies are attempting to develop treatments for MASLD/MASH (6). During the discovery and development of novel therapies, it is essential to have access to predictive animal models that sufficiently resemble the pathophysiology of human disease. In the field of MASH, rodent models are most frequently studied as they are often more practical to use than large animal models (7, 8). However, important differences between rodent and human physiology and metabolism support exploring non-rodent preclinical models of MASLD/MASH (9).

Pigs resemble human physiology and metabolism to a high degree as discussed elsewhere (10, 11). Göttingen Minipigs are a well-characterized and commonly used breed in drug development (12) and may also be a useful MASLD/MASH model. Because of their smaller size, they require much less test compound than farm pigs, and Göttingen Minipigs are still large enough to allow the collection of serial blood samples and liver biopsies across a study period. It was recently reported that Göttingen Minipigs fed a choline-deficient, amino-acid-defined, high-fat diet (CDAHFD) for 8 wk developed a MASH-like condition, with hepatic steatosis, inflammation, and perisinusoidal fibrosis in a chicken-wire pattern (11). However, it is important to know how liver pathology and hepatic fibrosis will continue to progress during a more extended feeding period and if the pathological changes induced by the CDAHFD in the livers of Göttingen Minipigs are reversible.

Therefore, the present study aimed to characterize the effect of a more extended feeding period with CDAHFD in Göttingen Minipigs, focusing on pathological changes in the liver, assessed by histology and RNA sequencing (RNAseq). Liver biopsies were collected up to four times from each minipig during the experimental phase, allowing for detailed examination of liver histopathology and global gene expression over time. At the same time points as the collection of liver biopsies, changes in liver stiffness were assessed noninvasively with shear wave elastography (SWE). An additional aim of the study was to explore whether the induced changes would regress during an 11-wk period where the diet for CDAHFD-fed minipigs was changed back to chow, in a manner similar to rodent studies (13, 14). Finally, a MASH Human Proximity Score based on gene-expression profiles, histology, and phenotype data was compared with human MASLD and MASH and to 41 rodent MASLD/MASH models, to estimate the translational value of CDAHFD-fed minipigs as a model of MASLD/MASH. (15)

## MATERIALS AND METHODS

### Animal Housing and Design of Animal Experiments

This study comprised two animal experiments performed under a license granted by the Danish Animal Experiments Inspectorate (license number: 2016-15-0201-01078). In both experiments, male Göttingen Minipigs (Ellegaard Göttingen Minipigs, Dalmose, Denmark) were castrated at 10–11 wk of age and gradually switched to their respective experimental diets over the following week. They were housed in groups according to the type of diet with straw as bedding material, various types of environmental enrichment, and unrestricted access to drinking water (nonacidified, nonchlorinated tap water). The room temperature was kept at 22–24°C. Lights were on between 6:00 AM and 7:00 PM and the minipigs were weighed twice weekly.

### Study 1

In the first pilot experiment (*study 1*), Göttingen Minipigs were fed twice daily with either chow (*n* = 12, Mini-Pig Expanded, Special Diets Services, Witham, UK) or CDAHFD (*n* = 16, Special Diets Services, Witham, UK), containing 1% cholesterol, 0.35% cholic acid, 30% fat (milk fat and cocoa butter), no choline, 0.1% methionine, and 20% fructose, supplemented with banana cream flavor (LorAnn Oils, Lansing, MI). After 4 mo on CDAHFD, 5 of the 16 Göttingen Minipigs became subdued and anorectic, and four of the five also were icteric. These five minipigs were therefore euthanized, and liver and plasma samples were collected. The remaining 11 minipigs on CDAHFD and the 12 chow-fed minipigs were euthanized a month later, after 5 mo on their respective diets. None of these minipigs showed any signs of disease. Blood samples were collected from awake and overnight fasted minipigs in EDTA-coated vacutainer tubes (Becton & Dickinson, Kongens Lyngby, Denmark) at the start of the experiment and after 2, 4, and, if applicable, 5 mo on the experimental diets. Right after the minipig was euthanized, the liver was dissected and weighed, and liver samples for histological analysis were collected from the left and right lateral and medial liver lobes.

### Study 2

**Figure 1.**
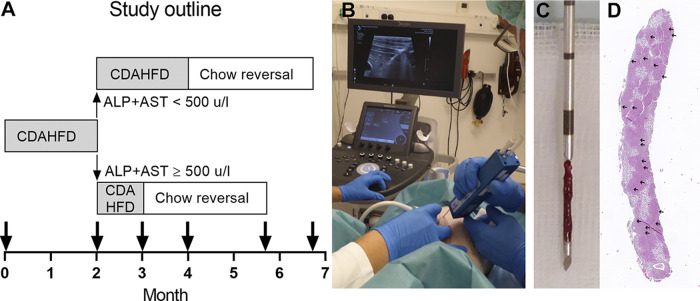
Design of *study 2*. *A*: Göttingen Minipigs were fed CDAHFD for 2 mo and based on the sum of plasma levels of ALP and AST then either allowed 1 or 2 mo more on CDAHFD before they were switched back to chow diet for 11 wk (same amount given to all pigs). The bold arrows indicate where plasma samples and liver biopsies/terminal liver samples were collected. At start of the experiment, only plasma samples were collected. *B*: liver biopsies were collected with ultrasound guidance. *C*: a 14-gauge Vacora vacuum-assisted biopsy system (Bard) was used for collection of liver biopsies. *D***:** H&E-stained section from an average-size liver biopsy, where 20 portal areas (marked with small arrows) could be identified.

*Study 2* was designed based on results from *study 1* where minipigs that were subdued at 4 mo tended to have higher levels of plasma alkaline phosphatase (ALP) and aspartate aminotransferase (AST) and developed more liver fibrosis than the other minipigs on CDAHFD (Supplemental Fig. S1, *A* and *B*). A significant and high level of correlation was observed between liver fibrosis at termination, and the sum of plasma ALP and AST measured after 2 mo on CDAHFD (Supplementary Fig. S1*C*). Therefore, based on the sum of the plasma concentration of ALP and AST measured after 2 mo on the CDAHFD, Göttingen Minipigs were then either allowed one (if ALP + AST ≥ 500 U/l) or two (if ALP + AST < 500 U/l) more months on CDAHFD before they were switched back to the chow diet. Both subgroups of CDAHFD-fed Göttingen Minipigs were given an 11-wk long chow reversal period (Fig. 1*A*). The Göttingen Minipigs fed chow from the start of the experiment remained on chow diet throughout the experimental period. Blood samples were collected from awake and overnight fasted minipigs, as indicated in Fig. 1*A*, in EDTA-coated vacutainer tubes and SST vacutainer tubes (both from Becton & Dickinson, Kongens Lyngby, Denmark). At the same time points, liver biopsies were collected; Göttingen Minipigs were first anesthetized by intramuscular injection of a mixture containing 0.25 mg/kg of butorphanol and 1.25 mg/kg of each of the following compounds: tiletamine, zolazepam, ketamine, and xylazine. Then, one or two liver biopsies (yielding a total minimum length of 1.3 cm) were collected (ultrasound guided) using a 14-gauge needle and a Vacora vacuum-assisted biopsy system (BARD Nordic, Helsingborg, Sweden) (Fig. 1, *B*–*D*). When the minipigs were euthanized, the liver was weighed, and liver samples for biochemical analysis and RNA extraction were collected from the left medial lobe. Liver samples for biochemical analysis were snap-frozen in liquid nitrogen, and liver samples for RNA extraction were stored in RNAlater at 4°C for 24 h, then the RNAlater was removed, and the samples were finally stored at −80°C. Liver samples for histology were collected from the left and right lateral and medial liver lobes.

Assessment of liver stiffness with two-dimensional shear wave elastography (SWE) was performed on minipigs anesthetized as detailed in the paragraph above and placed in dorsal recumbency using the Aixplorer ULTIMATE ultrasound system and an SL 10-2 transducer (SuperSonic Imagine, Aix-en-Provence, France). SWE was performed on all minipigs in the experiment at three-time points: *1*) right before the CDAHFD-fed minipigs were switched back to chow (t0), *2*) after 5.5 wk of chow reversal (t1), and *3*) after 11 wk chow reversal (t2), before euthanasia of the minipigs. An average (arithmetic mean) of three SWE measurements per minipig was used at each time point.

### Histology and Image Analysis

In both studies, liver biopsies collected during the experiment and liver samples collected right after euthanasia were fixed in 10% neutral buffered formalin (VWR International AB) for 4–5 days and subsequently embedded in paraffin. Sections of 3-µm thickness were then cut and stained with hematoxylin and eosin (HE, both chemicals from Sigma-Aldrich, Brøndby, Denmark) or Picrosirius Red (PSR; both chemicals from Sigma-Aldrich). In *study 2*, other sections were used for immunohistochemistry (IHC) with staining for CD45, Iba1, or smooth muscle actin (SMA), performed as described previously (11, 16) (detected with purple chromogen), except that the primary anti-SMA antibody was applied to tissue sections at a concentration of 0.5 µg/mL. As negative control, the primary antibodies were omitted from the staining procedures (see images of negative control stainings in Supplemental Fig. S2). After being mounted on cover glasses with Pertex (Sigma-Aldrich), all sections were scanned on a NanoZoomer 2.0 HT digital slide scanner (Hamamatsu, Hamamatsu City, Japan). Digital images of HE- and PSR-stained sections were used for semiquantitative scoring of the NAFLD activity score (evaluation of the parameters steatosis, lobular inflammation, and hepatocellular ballooning) and extent of fibrosis (evaluation of general hepatic fibrosis and perisinusoidal fibrosis) by expert liver pathologists (PB, DGT), as described previously (17). In addition, the extent of perisinusoidal fibrosis was scored on a semiquantitative scale (four levels). Image analysis of the digital image slides was performed with the software VIS (Visiopharm, Hørsholm, Denmark). The areas with positive staining for PSR, SMA, CD45, Iba1, or the area with steatosis in PSR-stained sections were detected with threshold-based image analysis applications and expressed as a percentage of the total area of a tissue sample (i.e., the proportional areas with steatosis, PSR, CD45, Iba1, and SMA staining). Furthermore, based on the quantified area with steatosis in each liver sample, the proportional areas with PSR, CD45, Iba1, and SMA staining of the nonsteatotic tissue area were calculated for each sample.

### Hematological Analysis of Blood Samples

In both studies, hematocrit (HCT) was measured in EDTA-stabilized blood samples on a Sysmex apparatus (Sysmex Corporation, Kobe, Japan) according to the manufacturer’s instructions.

### Analysis of Plasma Samples

In EDTA-stabilized plasma samples from both studies, concentrations of albumin, urea, creatinine, iron, bilirubin (total and conjugated), ALP, alanine aminotransferase (ALT), AST, γ-glutamyltransferase (GGT), glucose, phosphate, triglyceride, and cholesterol were measured on a Cobas 6000 Autoanalyser (Roche) according to the manufacturer’s instructions. Plasma concentration of insulin was measured as described previously (11).

### Biochemical Analysis of Tissue Samples

In *study 2*, liver samples for biochemical analysis were collected right after euthanasia and were snap-frozen in liquid nitrogen and stored at −80°C until analysis. The amounts of triglyceride, cholesterol, and glycogen were quantified in homogenized liver samples as described previously (17).

### RNA Extraction, Library Preparation, and Sequencing

In liver samples from *study 2*, RNA extraction was performed with RNeasy Mini Kit (Qiagen, Hilden, Germany) and library prepared with NEBNext Ultra II Directional RNA Library Prep Kit for Illumina (New England Biolabs, Ipswich, MA) in combination with unique dual indexing. QCs were performed on Fragment Analyzer (Agilent, Santa Clara, CA). Sequencing was performed on a NovaSeq 6000 with 150PE mode (Illumina, San Diego, CA).

### Processing and Analysis of RNAseq Data

The RNAseq data were processed and analyzed as shown in Supplemental Fig. S3. FastQC software v. 0.11.9 was used for quality control of individual FASTQ files (http://www.bioinformatics.babraham.ac.uk/projects/fastqc). RNA sequencing reads were aligned using hisat2 V2.1.0 (18) to the *Sus Scrofa* (pig) genome. The genes were counted using HTseq-count (v 0.11.1) (19). The raw gene-level counts were then used for differential gene expression analysis (Wald Test) using DESeq2 (20), an R-based open-source software developed to analyze transcriptomic data. The raw *P* values were adjusted by the Benjamini–Hochberg procedure to control the false discovery rate. Gene-expression in CDAHFD-fed minipigs was compared with chow-fed minipigs at two different time points: *1*) after 2 mo on CDAHFD, and *2*) right before initiation of the chow reversal period, where the disease had developed further, i.e., time point *t*0, where the minipigs had been fed CDAHFD for 3 or 4 mo (see Fig. 1*A*).

To assess for the enrichment of pathways, FGSEA package (21) was used for fast preranked gene set enrichment analysis. The analysis was applied to pathways from the KEGG database (22–24).

### Comparison and Ranking of Göttingen Minipig Model to Human MASLD/MASH and Rodent Models

The minipig model was scored according to the MASH Human Proximity Score (MHPS) as described previously (15). Briefly, MHPS evaluates models according to their translatability toward human MASLD/MASH and provides rankings based on metabolic significance or ability to induce MASH-fibrosis. This is facilitated through comparative analyses using transcriptomic, phenotypic and histopathological data generating the DSEA Human Proximity Score (DHPS), the Phenotype Human Proximity Score (PHPS), and the Human Histology Proximity Score (HHPS), respectively, which are averaged to give the final MHPS. The Phenotypic Homology Scoring System (PHPS) employed a 7-point scale to assess models based on human phenotypic outcomes, including body weight (BW), lipid profiles, liver-to-body weight ratio (LW/BW%), and liver enzyme levels (ALT and AST). HHPS incorporated qualitative assessments of human MASLD characteristics that murine models are expected to exhibit to histologically resemble human MASH. Meanwhile, the DHPS is an adaptation of the DSEA and “gene2drug” methods (25, 26), which ranks preclinical models based on their alignment with human RNA-seq results. For more details see the study by Vacca et al. (15) Human transcriptomics data as well as phenotype data from the study performed by Govaere et al. (27) was used. For the minipig model, the MHPS was calculated with two minor modifications applied to the PHPS scoring: First, points for increased ALT and AST levels between chow and CDAHFD fed animals were assigned based on a significant result of a two-sided Student’s *t* test as the number of animals did not suffice for a ROC curve analysis as described previously (15). Second, the minipig was assigned a point for increased LW/BW% based on data from the study by Pedersen et al. (11), where LW/BW% was increased threefold after 8 wk on CDAHFD, and data from *study 1*, where LW/BW% was also increased significantly threefold after 4 mo on CDA-HFD (data not shown). MHPS for the minipig model was finally compared with MHPS for 41 rodent models.

To explore in more detail how liver gene expression data revealed effects on hepatic fibrosis, expression of 30 key fibrosis-related genes previously identified in human MAFLD/MASH patients (28) were explored in the data set. In addition, expression of marker genes of inflammatory cell types in pigs identified in previous studies (29–32) were assessed, to further characterize hepatic inflammation.

### Statistical Analysis of In Vivo and Histology Data

Body weight data, data describing end points measured in plasma samples, and data describing biochemical end points in the liver samples were transformed with the natural logarithm and analyzed in a mixed model with diet-group and time point and the interaction between these factors as fixed effects and animal identity as a random effect (to account for repeated measurements in each minipig) using JMP version 14.1 (SAS Institute, Cary, NC) or SAS version 9.4 (SAS Institute). For each end point, the CDAHFD-fed group was compared with the chow-fed group at each relevant time point in *t* tests, with Bonferroni correction for multiple parallel comparisons (maximum four). Analysis of the histology data and the SWE data from *study 2* focused on the chow reversal period and included data from the following three time points: Right before chow reversal (= *t*0), after 5.5 wk of chow reversal (= *t*1), and after 11 wk chow reversal (= *t*2). Semiquantitative ordinal scoring data describing the pathological alterations were compared between chow-fed and CDAHFD-fed minipigs at t0 in a Fischer’s exact test (two-sided) using SAS, and between time points in CDAHFD-fed minipigs with the signed rank tests using SAS. Image analysis data and SWE data were analyzed in JMP in a mixed liner model, with diet-group and time point and the interaction between these factors as fixed effects and animal identity as a random effect. All data were transformed with the natural logarithm prior to analysis. Chow- and CDAHFD-fed minipigs were compared in this model at time point t0 with a *t* test, and the development in CDAHFD-fed minipigs were analyzed by comparison of time points *t*1 to *t*0 and *t*2 to *t*0 in two *t* tests, with Bonferroni correction for two parallel tests. The Spearman’s correlation coefficients between SWE and PSR proportional areas at *t*0, *t*1, and *t*2 were calculated with GraphPad Prism version 8 (GraphPad Software, San Diego, CA). Multivariate regression analysis of liver stiffness assessed by SWE in CDAHFD-fed minipigs as a function of the various quantitative histological end points and all pairwise interactions, again with animal identity as a random effect, was performed with JMP. Backward selection was applied for identification of significant explanatory variables. In all analyses, *P* values (adjusted where appropriate) <0.05 were considered statistically significant.

## RESULTS

### Main Findings in *Study 1*

**Figure 2.**
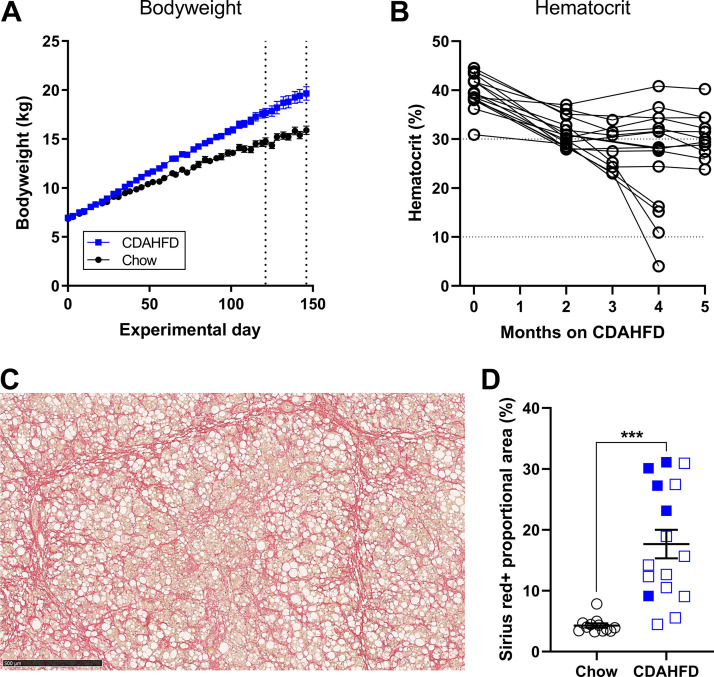
Body weight, hematocrit, and liver fibrosis in *study 1*. *A*: development in body weight. The vertical dotted lines indicate the time points 4 and 5 mo, where minipigs were euthanized. *B*: HCT levels for each individual CDAHFD-fed minipig in *study 1*. Four of the minipigs developed icterus after 4 mo on CDAHFD and had HCT levels <20%. *C*: PSR staining in liver section from one of the minipigs euthanized after 4 mo, to illustrate development of severe liver fibrosis. Scale bar = 500 µm. *D*: PSR proportional area in chow-fed and CDAHFD-fed minipigs, assessed in liver samples collected right after euthanasia. Closed blue symbols are observations from CDAHFD-fed minipigs euthanized after 4 mo. Open blue symbols are observations from CDAHFD-fed minipigs euthanized after 5 mo. Horizontal lines and error bars indicate means ± SE. ***P < 0.001 when the groups were compared.

Body weight was increased by ≈23% after 5 mo on CDAHFD compared with chow (*P* = 0.0012, Fig. 2*A*). Plasma concentrations of biomarkers indicative of liver damage (ALP, AST, and GTT) were significantly increased in CDAHFD-fed minipigs (Table 1). As described in the materials and methods, 5 of 16 minipigs in *study 1* were euthanized after 4 mo of CDAHFD feeding because they were subdued and anorectic and four of them had developed anemia (Fig. 2*B*). None of the remaining 11 minipigs on CDAHFD showed any clinical signs and were terminated after 5 mo on CDAHFD. Furthermore, the average plasma levels of bilirubin (total and conjugated bilirubin) were significantly increased by three- to sixfold in CDAHFD-fed minipigs after 2, 4, and 5 mo on CDAHFD (Table 1), and a nonsignificant trend toward decreased plasma levels of iron were also observed in minipigs on CDAHFD (Table 1). The minipigs on CDAHFD had developed severe liver fibrosis at euthanasia (Fig. 2*C*). Furthermore, the minipigs euthanized at 4 mo tended to have more fibrosis than the minipigs euthanized after 5 mo on CDAHFD (Fig. 2*D*).

**Table 1.** Plasma parameters measured in study 1

	Baseline		2 Mo		4 Mo		**5 Mo**†	
	Chow	CDAHFD	Chow	CDAHFD	Chow	CDAHFD	Chow	CDAHFD
Total protein, g/L	58.9 ± 0.8	61.4 ± 0.8	62.1 ± 0.6	62.3 ± 0.9	64.3 ± 1.0	58.9 ± 2.5	63.7 ± 0.7	65.7 ± 0.8
Albumin, g/L	40.7 ± 0.9	42.3 ± 0.7	48.5 ± 0.8	46.5 ± 1.1	51.5 ± 0.7	42.1 ± 2.0**	51.4 ± 0.6	43.8 ± 1.1**
Urea, mmol/L	1.5 ± 0.1	1.6 ± 0.1	1.9 ± 0.1	2.6 ± 0.1*	2.0 ± 0.1	2.6 ± 0.1*	2.3 ± 0.1	2.7 ± 0.1
Creatinine, µmol/L	67.7 ± 3.3	70.7 ± 2.0	80.1 ± 3.9	57.0 ± 2.0**	81.7 ± 4.1	51.8 ± 3.7***	76.4 ± 4.6	62.9 ± 2.7*
Iron, µmol/L	14.8 ± 1.1	16.3 ± 0.9	22.4 ± 1.2	18.7 ± 0.9	23.4 ± 1.2	20.7 ± 1.7	22.0 ± 0.9	17.9 ± 0.6
Total bilirubin, µmol/L	0.56 ± 0.10	0.64 ± 0.08	0.89 ± 0.12	3.50 ± 2.12**	0.94 ± 0.09	6.50 ± 10.31***	0.95 ± 0.08	3.21 ± 1.65**
Conjugated bilirubin, µmol/L	0.27 ± 0.05	0.25 ± 0.03	0.48 ± 0.06	1.90 ± 1.63***	0.50 ± 0.05	3.67 ± 4.82***	0.46 ± 0.03	1.59 ± 1.34**
ALP, U/L	29.2 ± 2.2	24.4 ± 3.7	20.3 ± 4.6	223.6 ± 68.1***	15.3 ± 5.1	330.9 ± 59.5***	15.8 ± 5.8	268.8 ± 93.1***
ALTL, U/L	39.1 ± 2.7	45.7 ± 2.2	66.3 ± 3.9	87.0 ± 8.8	60.4 ± 3.6	100.4 ± 10.0**	78.3 ± 4.9	101.1 ± 8.0
AST, U/L	41.4 ± 4.5	40.2 ± 4.3	29.8 ± 3.1	173.1 ± 41.0***	29.2 ± 2.7	306.7 ± 99.3***	53.6 ± 6.6	299.0 ± 68.8***
GGT, U/L	233.4 ± 26.5	227.6 ± 35.2	177.3 ± 22.4	459.4 ± 46.8**	89.4 ± 8.6	287.3 ± 105.6***	377.5 ± 61.1	1425.2 ± 136.4***
Phosphate, mmol/L	2.1 ± 0.1	2.3 ± 0.04	2.3 ± 0.04	2.3 ± 0.04	2.1 ± 0.03	2.3 ± 0.06	2.2 ± 0.03	2.3 ± 0.04
Triglyceride, mmol/L	0.49 ± 0.02	0.54 ± 0.03	0.60 ± 0.03	0.73 ± 0.10	0.63 ± 0.03	0.92 ± 0.27*	0.50 ± 0.03	0.81 ± 0.11*
Insulin, pmol/L, and glucose§	62.3 ± 13.3	61.9 ± 15.8	21.8 ± 6.9	34.5 ± 8.0	23.0 ± 4.4	38.2 ± 11.3	39.5 ± 9.5	39.6 ± 6.5
Cholesterol, mmol/L	1.8 ± 0.1	1.9 ± 0.1	1.9 ± 0.1	8.6 ± 1.3***	2.1 ± 0.1	9.5 ± 1.3***	2.1 ± 0.1	11.0 ± 1.2***

Values are mean plasma concentration ± SE. *, **, and *** indicate *P* < 0.05, 0.001, and 0.0001, respectively, when CDAHFD was compared with chow at each time point. †Mean values calculated only for those animals that were euthanized at 5 mo. §Plasma glucose concentrations at termination were similar in controls (4.8 ± 0.1 mmol/L) and CDAHFD-fed minipigs (4.8 ± 0.2 mmol/L).

### Body Weight and Hematological Parameters in *Study 2*

**Figure 3.**
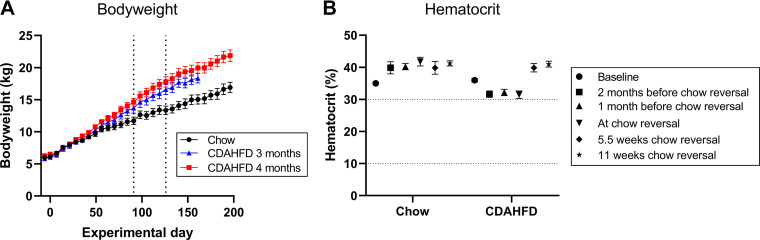
Body weight and hematocrit in *study 2*. *A*: development in body weight. The vertical dotted lines indicate the time points 3 and 4 mo, respectively, where CDAHFD-fed pigs were switched back to chow diet. *B*: mean HCT levels in *study 2*. Mean HCT was decreased slightly during the period the minipigs were fed CDAHFD but was restored to levels similar to chow-fed minipigs during the chow reversal period. Error bars indicate the SE.

In *study 2*, the Göttingen Minipigs fed CDAHFD also gained more body weight than minipigs on chow (Fig. 3*A*). Bodyweight was increased by ≈22% (*P* = 0.003) and ≈36% (*P* = 0.0008) after 3 and 4 mo on CDAHFD, respectively (Fig. 3*A*). Interestingly, at the end of the chow reversal period bodyweight of the minipigs initially fed CDAHFD was still increased by ≈29% (*P* = 0.0006) compared with the minipigs which were fed chow throughout the entire study period (Fig. 3*A*).

No minipigs on CDAHFD in *study 2* became icteric, and while a trend toward a decreased average HCT was observed during the CDAHFD-feeding period, the average HCT in the CDAHFD-group never decreased below 30%, and during the chow reversal period, the average HCT was restored to the level found in chow-fed minipigs (Fig. 3*B*). Plasma levels of conjugated bilirubin were increased significantly (≈3- to 4-fold) at *t*0, i.e., after 3 or 4 mo on CDAHFD, but at the end of the chow reversal period, plasma levels of total and conjugated bilirubin were comparable to chow-fed minipigs (Table 2).

**Table 2.** Plasma and liver parameters measured in study 2

	Baseline		2 Mo		Time Point *t*0 (3 or 4 Mo on CDAHFD)		End of Study	
	Chow	CDAHFD	Chow	CDAHFD	Chow	CDAHFD	Chow	CDAHFD + 11 wk chow reversal
Total protein, g/L	61.6 ± 1.4	59.9 ± 0.7	63.6 ± 0.9	60.6 ± 0.8	65.4 ± 0.4	64.6 ± 1.1	68.4 ± 0.6	68.6 ± 0.8
Albumin, g/L	39.6 ± 1.2	40.5 ± 0.5	48.8 ± 0.6	45.0 ± 1.0	52.0 ± 0.9	46.0 ± 1.3*	52.7 ± 0.8	50.0 ± 0.8
Urea, mmol/L	1.6 ± 0.2	1.4 ± 0.1	2.2 ± 0.1	4.3 ± 0.2***	2.2 ± 0.2	2.3 ± 0.1*	2.1 ± 0.1	2.0 ± 0.2
Creatinine, µmol/L	62 ± 3	62 ± 1	67 ± 2	54 ± 1**	76 ± 3	60 ± 2**	75 ± 4	82 ± 3
Iron, µmol/L	9.5 ± 1.0	12.0 ± 0.7*	22.6 ± 0.7	19.0 ± 0.6	21.3 ± 2.8†	14.9 ± 1.3†	21.7 ± 1.2	20.2 ± 0.7
Total bilirubin, µmol/L	0.44 ± 0.12	0.76 ± 0.11	0.55 ± 0.12	0.63 ± 0.19	0.87 ± 0.29	3.02 ± 1.30***	0.53 ± 0.11	0.67 ± 0.08
Conjugated bilirubin, µmol/L	0.23 ± 0.04	0.37 ± 0.06	0.32 ± 0.05	0.86 ± 0.16**	0.41 ± 0.16	0.97 ± 0.58*	0.38 ± 0.05	0.34 ± 0.06
ALP, U/L	25.3 ± 4.7	20.0 ± 5.3	32.3 ± 7.7	233.4 ± 59.7***	29.1 ± 11.7†	382.5 ± 82.7***†	16.3 ± 6.5	15.5 ± 5.4
ALT, U/L	30.6 ± 4.6	39.0 ± 1.8	74.6 ± 3.5	61.4 ± 4.8	75.1 ± 3.1	100.6 ± 8.4	78.3 ± 3.5	60.8 ± 2.9
AST, U/L	37.7 ± 5.2	36.4 ± 4.6	28.7 ± 1.8	77.6 ± 15.9***	30.5 ± 3.0	192.0 ± 61.5***	36.9 ± 4.4	48.2 ± 6.9
GGT, U/L	227.5 ± 53.1	175.6 ± 33.6	99.1 ± 9.3	130.7 ± 23.8	173.3 ± 64.6†	335.4 ± 44.1†	185.5 ± 30.4	175.9 ± 19.5
Glucose, mmol/L	4.9 ± 0.1	5.2 ± 0.2	5.1 ± 0.1	5.7 ± 0.2	4.6 ± 0.2	4.5 ± 0.1	4.1 ± 0.1	4.4 ± 0.1
Insulin, pmol/L	27.4 ± 12.1	42.0 ± 6.21	71.2 ± 11.6	128.0 ± 18.7	71.0 ± 22.7	46.0 ± 4.7	37.2 ± 8.9	84.2 ± 10.6*
Phosphate, mmol/L	2.7 ± 0.1	2.6 ± 0.1	2.3 ± 0.1	2.6 ± 0.1*	2.4 ± 0.03†	2.3 ± 0.03†	2.1 ± 0.04	2.2 ± 0.05
Triglyceride, mmol/L	0.45 ± 0.04	0.37 ± 0.02	0.41 ± 0.04	0.64 ± 0.13*	0.52 ± 0.04	0.83 ± 0.08*	0.42 ± 0.01	0.38 ± 0.03
Cholesterol, mmol/L	2.1 ± 0.1	2.2 ± 0.1	1.7 ± 0.1	7.4 ± 1.0***	1.7 ± 0.1	12.2 ± 1.1***	1.7 ± 0.1	1.9 ± 0.1
Liver								
Glycogen, µmol/g tissue							203.7 ± 36.7	128.6 ± 14.9*
Triglyceride, µmol/g tissue							7.6 ± 0.9	12.0 ± 2.1
Cholesterol, µmol/g tissue							5.5 ± 0.5	17.3 ± 1.7***

Values are mean plasma concentration ± SE. *, **, and *** indicate *P* < 0.05, 0.001, and 0.0001, respectively, when CDAHFD was compared with chow at each time point. †Geometric mean values calculated on *n* = 3 chow animals and *n* = 5 CDAHFD animals.

### Göttingen Minipigs Fed CDAHFD Developed a MASH-like Liver Histology with Hepatic Steatosis, Hepatocyte Ballooning, Lobular Inflammation, and Pronounced Fibrosis

**Figure 4.**
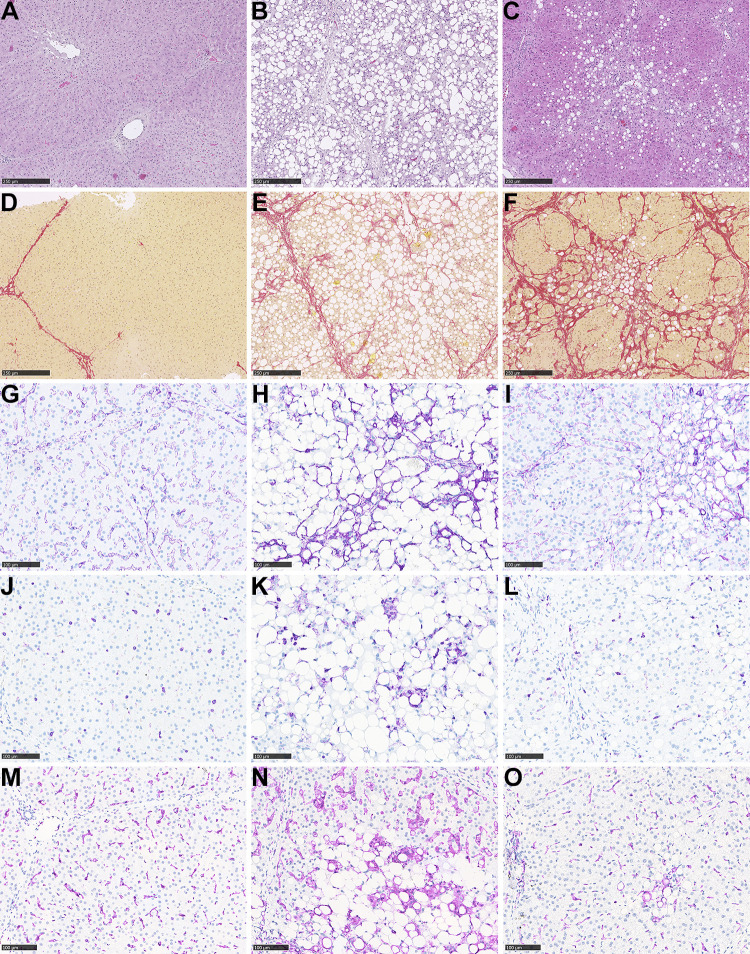
Histology in Göttingen Minipig livers. HE-stained liver sections, from a chow-fed control minipig (*A*) and a CDAHFD-fed minipig before chow reversal (*B*) and after 11 wk of chow reversal (*C*) (*B* and *C* are from the same minipig, scale bar = 250 µm). PSR-stained liver sections, from a chow-fed control minipig (*D*) and a CDAHFD-fed minipig before chow reversal (*E*) and after 11 wk of chow reversal (*F*) (*E* and *F* are from the same minipig, scale bar = 250 µm). Liver sections stained for SMA from a chow-fed control minipig (*G*) and a CDAHFD-fed minipig before chow reversal (*H*) and after 11 wk of chow reversal (*I*) (*H* and *I* are from the same minipig, scale bar = 100 µm). Liver sections stained for CD45, from a chow-fed control minipig (*J*) and a CDAHFD-fed minipig before chow reversal (*K*) and after 11 wk of chow reversal (*L*) (*K* and *L* are from the same minipig, scale bar = 100 µm). Liver sections stained for Iba1 from a chow-fed control minipig (*M*) and a CDAHFD-fed minipig before chow reversal (*N*) and after 11 wk of chow reversal (*O*) (*N* and *O* are from the same minipig, scale bar = 100 µm).

**Figure 5.**
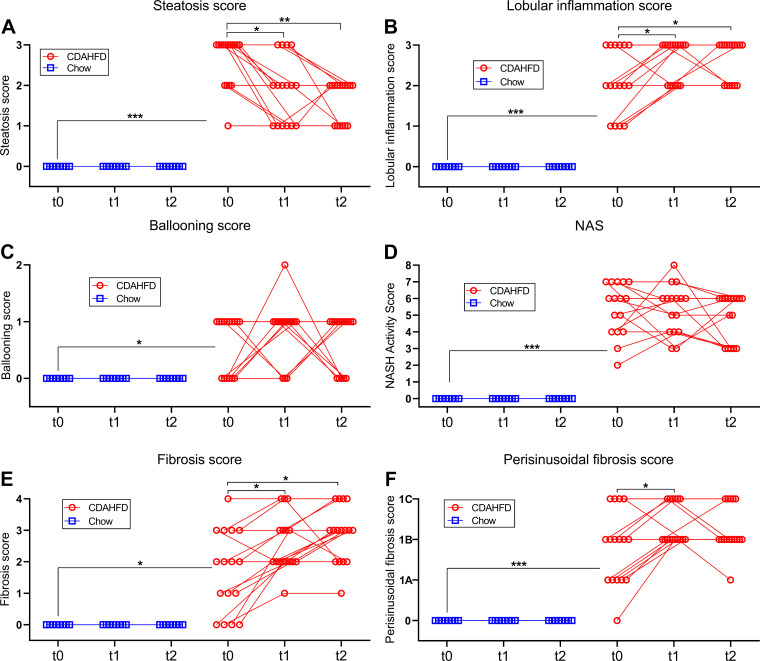
MASH activity score and fibrosis score of liver samples collected in *study 2* from start to end of the chow reversal period. *A*: steatosis score. *B*: lobular inflammation score. *C*: ballooning score. *D*: NAFLD activity score. *E*: fibrosis score. *F*: perisinusoidal fibrosis score. On all panels *t*0 = the biopsy collected right before start of the chow reversal period, *t*1 = the biopsy collected after 5.5 wk chow reversal and *t*2 = the terminal sample collected after 11 wk chow reversal. Symbols indicate observations from an individual minipig and observations from the same animal are connected with lines. *, **, and *** indicate *P* < 0.05, 0.001, and 0.0001, respectively, in the indicated comparisons.

**Figure 6.**
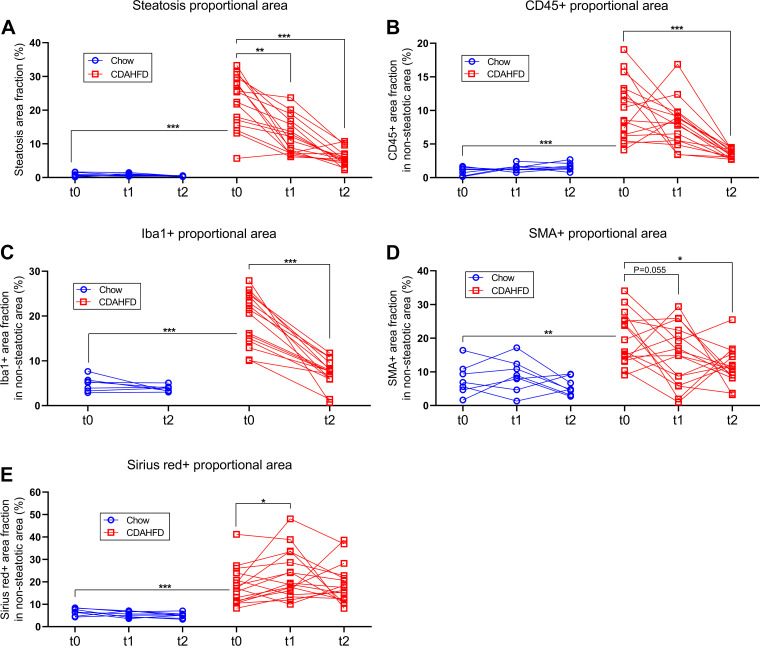
Image analysis results from liver samples collected in *study 2* from start to end of the chow reversal period. *A*: proportional area of the liver tissue with steatosis. *B*: proportional nonsteatotic area of the liver tissue positive for CD45. *C*: proportional nonsteatotic area of the liver tissue positive for Iba1. *D*: proportional nonsteatotic area of the liver tissue positive for SMA. *E*: proportional nonsteatotic area of the liver tissue positive for Picrosirius Red. On all panels *t*0 = the biopsy collected right before start of the chow reversal period, *t*1 = the biopsy collected after 5.5 wk chow reversal, and *t*2 = the terminal sample collected after 11 wk chow reversal. Symbols indicate observations from an individual minipig and observations from the same animal are connected with lines. *, **, and *** indicate *P* < 0.05, 0.001, and 0.0001, respectively, in the indicated comparisons.

Representative pictures of liver sections from *study 2* stained with H&E, PSR, or by IHC for CD45, Iba1 or SMA are shown in Fig. 4. Minipigs on CDAHFD developed pronounced hepatic steatosis (macrovesicular type), and hepatocyte ballooning and lobular inflammatory foci (Fig. 4, *B* and *C*). Hepatic fibrosis (i.e., clearly distinguishable from the normal interlobular connective tissue septae seen in porcine liver) occurred as portal/periportal and perisinusoidal fibrosis, which was found in a chicken-wire pattern and some minipigs (5 of 16) progressed to cirrhosis with portal-central fibrous septa and distortion of lobular architecture (Fig. 4, *E* and *F*). Evaluation of MASLD activity using NAS showed a significant increase in the individual histological parameters: steatosis score, lobular inflammation score and ballooning score, as well as in the composite NAS in minipigs fed CDAHFD for 3 or 4 mo compared with chow-fed minipigs (Fig. 5, *A*–*D*). Furthermore, the overall fibrosis and perisinusoidal fibrosis scores were also significantly increased in the CDAHFD-group compared with chow (Fig. 5, *E* and *F*). Notably, 15 of 16 CDAHFD-fed minipigs developed minimum fibrosis score 2, and, as mentioned above, several minipigs developed cirrhosis (i.e., fibrosis score 4) during this relatively short experiment. In agreement with the semiquantitative scoring data, the proportional areas of the liver tissue with steatosis and positive staining for PSR were also significantly increased in minipigs on CDAHFD for 3 or 4 mo (Fig. 6, *A* and *E*).

IHC staining for CD45 and Iba1 revealed an increase in single-positive cells (resident Kupffer cells and infiltrating leukocytes) and positive cells in foci and clusters and highlighted lipogranulomas in minipigs fed CDAHFD (Fig. 4, *K* and *N*). Furthermore, immunostaining for SMA, a marker of activated hepatic stellate cells, was also increased in CDAHFD-fed minipigs, and was observed in the space of Disse along the sinusoids and sometimes surrounding steatotic hepatocytes (Fig. 4, *H* and *I*). Quantification of the proportional areas with positive staining for CD45, Iba1, and SMA revealed a significant increase in CDAHFD-fed minipigs compared with minipigs on chow (Fig. 6, *B*–*D*).

Plasma concentrations of biomarkers indicative of liver damage (ALP, AST, and GTT) were significantly increased in CDAHFD-fed minipigs in *study 2* (Table 2). Furthermore, the increased levels of urea and the statistically significantly decreased levels of creatinine (Table 2) has been seen to reflect impaired liver function (33, 34). Plasma glucose was comparable in chow and CDAHFD-fed minipigs (Table 2), and plasma insulin levels displayed nonsystematic fluctuations across time points and groups (Table 2, see also Table 1). Plasma cholesterol was increased up to approximately sixfold in minipigs on CDAHFD (Table 2), and plasma levels of triglycerides were increased with ≈50% in CDAHFD-fed minipigs (Table 2).

### The Liver Gene-Expression Profile, Phenotypical and Histopathological Data Revealed CDAHFD-Fed Minipigs Have High Resemblance to Human MASLD/MASH

**Figure 7.**
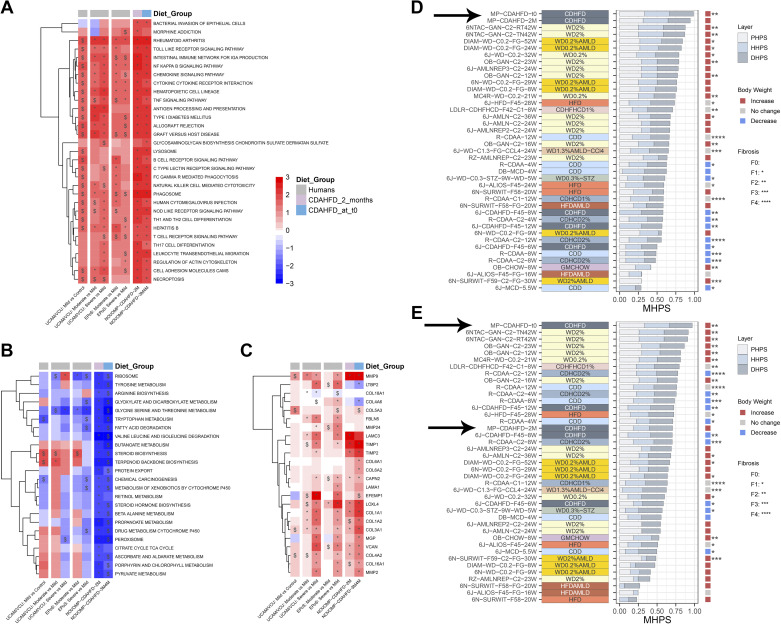
Hepatic gene-expression differences between CDAHFD-fed and chow-fed Göttingen Minipigs. *A*: 30 KEGG pathways identified with significantly increased activity and normalized enrichment score >2. *B*: 24 KEGG pathways identified with significant decreased activity and normalized enrichment score <-2. *C*: expression of 24 genes (identified in Ref. 28) involved in fibrosis in human MAFLD/MASH in CDAHFD-fed minipigs and in human MASLD/MASH patients. *D*: comparison and ranking of MHPS in CDAHFD-fed minipigs (time points: 2 mo and t0, marked with an arrow) and 41 rodent models relative to disease characteristics in MASLD/MASH patients with predominantly metabolic alterations. *E*: comparison and ranking of MHPS in CDAHFD-fed minipigs (time points: 2 mo and *t*0, both are marked with arrows) and 41 rodent models relative to disease characteristics in human MASLD/MASH patients characterized by more advanced disease and fibrosis.*, **, and *** indicate adjusted *P* value <0.05, <0.01, and <0.001, respectively, vs. chow-fed minipigs and $ *P* value <0.05 vs. chow-fed minipigs. KEGG pathways (*A* and *B*) were clustered according to the Ward method. NAFLD activity scores and fibrosis scores for the 2-mo time point in comparison to the *t*0 time point are shown in Supplemental Fig. S6.

All differentially expressed genes in CDAHFD-fed minipigs compared with chow-fed minipigs after 2 mo and at *t*0 (i.e., right before initiation of chow reversal) are listed in Supplemental Table S2 and S3, respectively, and pathways which were significantly up- or downregulated and with a numerical normalized enrichment score >2, identified in the pathway enrichment analysis with the KEGG database, are shown in Fig. 7, *A* and *B*. Overall, pathway regulation appeared fully comparable in CDAHFD-fed minipigs after 2 mo on CDAHFD and at time point *t*0, and had high resemblance to regulated pathways in human MASH. Multiple pathways, including several involved in inflammation, were significantly upregulated, while several of the downregulated pathways were involved in amino acid metabolism and steroid biosynthesis (Fig. 7, *A* and *B*). Pathway regulation in minipigs, 41 rodent models, and human MASH patients is shown in Supplemental Fig. S4.

Of 30 key fibrosis-related genes in human MASLD/MASH (28), 24 genes were identified in RNAseq data from minipig liver (Fig. 7*C*). Of these 24 genes, 12 were expressed significantly different in CDAHFD-fed minipig after 2 mo, whereas 19 of 24 genes had significantly altered expression at *t*0 (Fig. 7*C*). Additionally, expression levels of marker genes of inflammatory cells indicated that macrophages and various T cell populations were increased in the livers of CDAHFD-fed minipigs at t0 (Supplemental Fig. S5).

The overall translatability of the minipig model toward human MASLD and MASH was further evaluated by comparative analysis of the transcriptomic, phenotypic, and histopathological data, to generate the MHPS as described previously (15), which was then compared with the MHPS scores for 41 rodent models of MASLD/MASH (15). Figure 7*D* shows the ranking of minipigs and rodent models when compared with disease characteristics in MASLD/MASH patients with predominantly metabolic alterations. Minipigs fed the CDAHFD for the longest duration (3 or 4 mo; *t*0) were ranked first and those fed CDAHFD for two months were ranked second (Fig. 7*D*). MHPS in the minipig and rodent models were also compared with disease characteristics in human MASLD/MASH patients characterized by more advanced fibrosis (Fig. 7*E*). Here CDAHFD-fed minipigs at *t*0 were ranked as number 1, whereas the intermediate 2-mo time point was ranked as number 16. This difference in ranking was in agreement with the liver histology data, which showed that CDAHFD-fed minipigs at time point *t*0 had developed considerably more fibrosis than at the 2-mo group (Supplemental Fig. S6).

### Liver Fibrosis Did Not Regress during an 11-wk Reversal Period

During an 11-wk chow reversal period it was explored to what extent histological alterations in the liver induced by the CDAHFD would regress. The proportional areas of steatosis, CD45-, Iba1-, and SMA-positive staining decreased significantly by ≈75% (*P* < 0.0001), ≈65% (*P* < 0.0001), ≈65% (*P* < 0.001) and ≈45% (*P* = 0.0004), respectively (Fig. 6, *A-D*). In agreement with this, the steatosis score decreased significantly during the chow reversal period (Fig. 5*A*). However, the lobular inflammation score, the fibrosis score, and perisinusoidal fibrosis score increased significantly during the chow reversal period (*P* = 0.0078, *P* = 0.0034, and *P* = 0.0078, respectively, Fig. 5, *B*, *E* and *F*), the ballooning score remained unchanged (Fig. 5*C*) and the PSR-positive proportional area was also not significantly decreased (Fig. 6*E*).

Biochemical analysis of the liver tissue revealed increased levels of cholesterol (*P* < 0.0001, Table 2), decreased levels of glycogen (*P* = 0.0366, Table 2), and a nonsignificant trend toward increased levels of triglycerides (*P* = 0.0615, Table 2) at the end of the chow reversal period.

In plasma, concentrations of the enzymes ALP, AST, GGT, and the levels of total cholesterol and triglyceride also decreased during the chow reversal period and were no longer significantly elevated in the minipigs previously fed with CDAHFD (Table 2).

### Liver Stiffness Assessed with Shear Wave Elastography Was Dependent on Fibrosis as Well as Steatosis and Infiltration with CD45-Positive Cells

**Figure 8.**
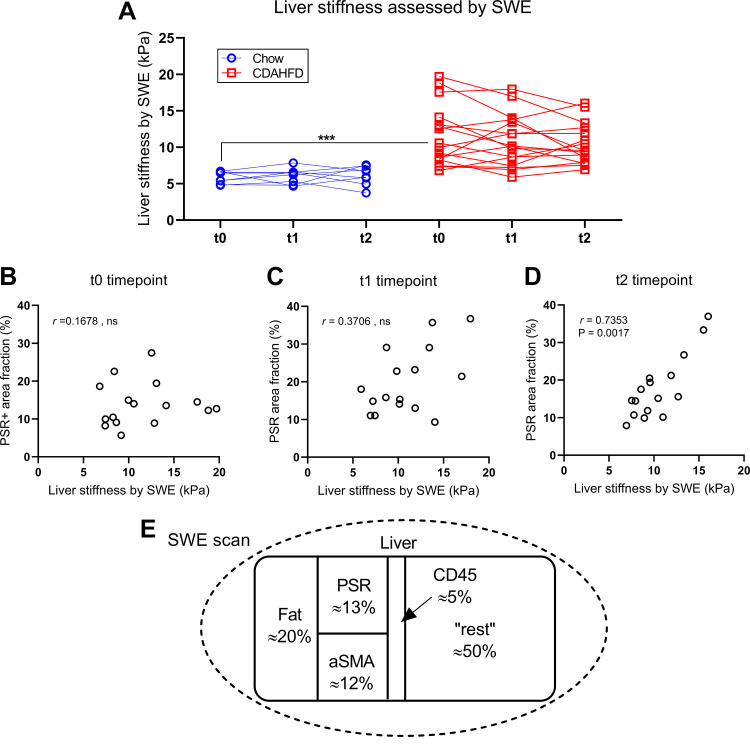
Liver stiffness assessed by SWE and correlation to fibrosis. *A*: liver stiffness assessed with SWE from start to end of the chow reversal period in *study 2*. *B*: correlation between the proportional area of the liver tissue with Picrosirius Red staining and liver stiffness assessed with SWE right before chow reversal. *C*: correlation between the proportional area of the liver tissue with PSR staining and liver stiffness assessed with SWE after 5.5 wk chow reversal. *D*: correlation between the proportional area of the liver tissue with PSR staining and liver stiffness assessed with SWE after 11 wk chow reversal. *E*: schematic representation of the liver tissue at the start of the chow reversal period. SWE was used to estimate the overall liver stiffness and with histology and image analysis the liver content of steatosis, fibrosis (PSR), SMA, and CD45+ infiltration was estimated. Multivariate regression analysis was then used to explore how these components individually influenced liver stiffness. *t*0, the biopsy collected right before start of the chow reversal period; *t*1, the biopsy collected after 5.5 wk chow reversal; *t*2, the terminal sample collected after 11 wk chow reversal. Symbols indicate observations from an individual minipig and observations from the same animal are connected with lines. ***Indicates *P* < 0.0001 when chow-fed and CDAHFD-fed minipigs were compared as indicated. ns: not significant.

Shear wave elastography was used for quantification of liver stiffness and was significantly increased in CDAHFD-fed minipigs at the time of initiation of chow reversal compared with chow-fed minipigs (Fig. 8*A*, P < 0.0001) but did not decrease significantly during the chow reversal period (Fig. 8*A*). Furthermore, liver stiffness assessed with SWE and the proportional area of the liver with positive staining for PSR was only significantly correlated at the last time point in the chow reversal period in CDAHFD-fed minipigs (i.e., at termination of the experiment) (Fig. 8, *B*–*D*). This led us to perform a multivariate regression analysis between liver stiffness assessed by SWE and the proportional areas with steatosis, fibrosis, CD45-, and SMA-positive staining, which had been assessed by image analysis and which markedly changed over the chow reversal period (Fig. 8*E*). This revealed that liver stiffness assessed by SWE was significantly dependent on the proportional areas with fibrosis, steatosis, and CD45-positive cells (Table 3). Increasing amounts of fibrosis and steatosis both contributed with increased liver stiffness, whereas increasing amounts of CD45-positive cells contributed with decreased liver stiffness (Table 3).

**Table 3.** Parameter estimates from analysis of SWE by multiple linear regression

Parameter	Estimate	*P* Value
Steatosis proportional area	0.193	0.0019
PSR-positive proportional area	0.207	0.0321
CD45-positive proportional area	−0.227	0.0040

## DISCUSSION

The main findings in this study are that Göttingen Minipigs fed CDAHFD developed a MASH-like phenotype, with hepatic steatosis, inflammation, hepatocyte ballooning, and considerable fibrosis, which progressed to cirrhosis (i.e., F4) in several minipigs. In addition, plasma levels of ALP, AST, GGT, cholesterol and urea were increased, and plasma levels of creatinine were decreased. Combined evaluation of gene expression data, key phenotypic parameters, and histopathological alterations benchmarked against human MASLD and MASH patients (i.e., the MHPS), revealed that CDAHFD-fed minipigs ranked high among the different preclinical models evaluated. When MHPS for the minipig model was compared with disease characteristics from human MASLD/MASH patients characterized by advanced fibrosis, there was a clear difference between the 2-mo time point (ranked as number 16) and the *t*0 time point (ranked as number one). This reflects that there was considerable progression of the fibrosis from the 2-mo time point to the *t*0 time point. Overall, these findings show that CDAHFD-fed Göttingen Minipigs is a promising model of human MASLD/MASH.

To gain an initial understanding of the potential for disease regression in this model, all minipigs receiving CDAHFD were switched to receive chow diet for 11 wk starting at *t*0. The 11-wk chow reversal period resulted in near-complete regression of hepatic steatosis; however, lobular inflammation was increased over this same period resulting no change in the composite MASH activity score (albeit the CD45 proportional area decreased markedly, see further discussion below). The fibrosis and perisinusoidal fibrosis scores were increased significantly during the chow reversal period. In a recent study conducted in the same model, but at an earlier stage, obeticholic acid was shown to reduce fibrosis, without effect on MASH progression, mimicking findings in human patients (10). This documents that clinically documented antifibrotic effects can be captured in the model. In MASH patients it is known that efficient life-style intervention, which results in a body weight loss, can decrease hepatic steatosis and inflammation (35). There is also evidence that hepatic fibrosis can regress after a more extended period of pharmacological intervention (36) or after pronounced body weight loss (37).

Regression of histological alterations will likely depend on the duration of an intervention. A previous study with CDAHFD-fed mice reported that steatosis had regressed completely after 4 wk of chow reversal, and while inflammation had regressed entirely after 12 wk of chow reversal, fibrosis was still present at this time point, with little signs of regression (14). In another study with mice on a Western diet, steatosis and inflammation regressed after 7 wk of chow reversal, and a significant reduction of portal fibrosis was observed after 12 wk, whereas the perisinusoidal fibrosis persisted after 16 wk of chow reversal (13). The 12-wk chow reversal period in the mouse studies represents a relatively longer part of the expected lifetime of this species than the 11-wk chow reversal period used in the present minipig study.

Pathology scoring of liver sections was complemented by image-analysis based quantification of steatosis, PSR, CD45, Iba1, and SMA staining. Consistent with the reduction in steatosis score, the area with steatosis was reduced in the CDAHFD group over the 11-wk chow reversal period. While PSR area was unchanged at the end of the 11-wk period, infiltration with CD45-, Iba1-, and SMA-positive cells (marker for activated hepatic stellate cells responsible for fibrogenesis) were significantly decreased. This ∼65% reduction in CD45- as well as Iba1-positive proportional area may be an early indicator of improved pathology that, with a longer intervention period, would result in a reduction in lobular inflammation scoring. In that context, it should be noted that the lobular inflammation score is a semiquantitative evaluation of the number of inflammatory foci per liver lobule, which neither takes the potential size changes of the foci into account, nor the number of inflammatory cells that are not found in foci.

In *study 2*, SWE was explored as a potential noninvasive indicator of liver fibrosis in Göttingen Minipigs. Liver stiffness was increased in CDAHFD-fed minipigs, with values similar to those found in MASH patients (38, 39). Liver stiffness however was unchanged during the chow reversal period. This is in line with the unchanged PSR proportional area but in contrast to the observed increases in fibrosis score over this time period. It has previously been reported that SWE can be used for prediction of fibrosis in patients with MASLD. However, it is important to acknowledge that factors other than fibrosis can influence liver stiffness. Steatosis grade has been reported to influence the diagnostic performance of noninvasive assessment of fibrosis tests in patients with MASLD (40) as well as patients with chronic hepatitis (41). However, how steatosis impacts liver stiffness is somewhat controversial as both reductions (39) and increases (41) in liver stiffness have been attributed to the presence of steatosis. Inflammation has also been reported to influence diagnostic performance regarding liver stiffness (42, 43). Interestingly, a study performed with different rat models of MASLD reported that inflammation score in multivariate regression analysis was negatively correlated with liver stiffness assessed by SWE (44). We observed a similar effect of inflammation, expressed as the CD45-positive proportional area (Table 3). Therefore, SWE appears most useful for noninvasive assessment of liver fibrosis in minipigs in situations where other parameters that can influence liver stiffness are absent or do not vary between the animals.

An advantage of Göttingen Minipigs as a MASH-model compared with rodents is the ability to repeatedly collect liver biopsies and sizeable blood samples from each animal during an experiment. The liver biopsies collected in this study were fully comparable to the recommended size of liver biopsies collected from human MASH patients (45). Furthermore, the procedure, where up to four liver biopsies were collected from each minipig within a period of ≈3.5 mo (Fig. 1), was well tolerated by all animals. Another advantage of Göttingen Minipigs is that rodents fed a CDAHFD do not typically become obese, while a significant increase in body weight was observed in our studies and in a comparable recent study (10). However, as choline deficiency blocks key metabolic pathways, and acknowledging that the model did not have clear metabolic dysfunction or noticeable changes in insulin sensitivity, this model likely mainly has potential for targets with anti-inflammatory and antifibrotic mechanisms.

A challenge that we identified with the CDAHFD-fed minipig model is that long exposure to CDAHFD can result in severe anemia. In *study 1*, four of 16 minipigs developed icterus and anemia after exposure to CDAHFD for 4 mo. These effects were most likely caused by the choline-deficient diet. Decades ago, it was reported that choline-deficient diets resulted in anemia in rats, dogs, and piglets (46–48). In mice on CDAHFD, it is not known if anemia develops over time. It is also unknown how hemolysis might influence disease development and translatability of models based on CDAHFD. In that connection, it should be noted that in humans with MASH, increased rather than decreased hematocrit has been found, and hematocrit further was found to correlate with the severity of the disease (49). Encouragingly, the algorithm that we developed and applied after two months of CDAHFD feeding to evaluate disease progression mitigated the anemia and resulted in a more homogenous study population at the end of study. Another challenge with porcine models is that the normal liver contains fibrous septae between the liver lobules. These septae are not seen in human liver and needs to be considered when fibrosis is assessed. However, the fibrosis found in CDAHFD-fed minipigs was clearly discernible from the normal interlobular connective tissue septae.

In conclusion, we have shown that CDAHFD-fed minipigs develop a MASH-like phenotype which in several aspects resemble the changes observed in human patients with MASLD/MASH. It was possible to closely monitor disease development by collecting serial liver biopsies from each animal. However, Göttingen Minipigs can develop anemia if they are maintained on CDAHFD for longer than 3 mo, and this must be considered when studies are designed. During an 11-wk chow reversal period, the steatosis was markedly reduced, but other pathological alterations did not clearly regress. Still, a recent 12-wk study in this model showed that treatment with obeticholic acid, initiated after 4 wk on CDAHFD, could prevent development of fibrosis (10). Future studies are needed to demonstrate how the model can best be used to evaluate effects of therapeutic interventions that are currently being developed.

## DATA AVAILABILITY

Source data for this study are not publicly available due to privacy restrictions. The source data are available to verified researchers upon request by contacting the corresponding author.

## SUPPLEMENTAL MATERIAL

10.5281/zenodo.12662559 Supplemental Tables S2 and S3 and Supplemental Figs. S1–S6: https://zenodo.org/doi/10.5281/zenodo.12662559.

## GRANTS

This study has been conducted as part of the preclinical work package of the Liver Investigation: Testing Marker Utility in Steatohepatitis (LITMUS) project. The LITMUS study is a large multicenter study aiming to evaluate Non-Alcoholic Fatty Liver Disease biomarkers. The Innovative Medicines Initiative 2 (IMI2) Joint Undertaking under Grant Agreement 777377 funded the LITMUS study. This Joint Undertaking receives support from the European Union’s Horizon 2020 research and innovation program and EFPIA.

## DISCLAIMERS

The content is solely the authors responsibility and does not necessarily reflect the official views of the institutions they are affiliated with.

## DISCLOSURES

The authors H.H., S.T.H., L.M.H., R.K.K., M.L., L.F.M., and H.D.P. own minor stock portions in Novo Nordisk A/S and JWP own a minor stock portion in Eli Lilly and Company. None of the other authors has any conflicts of interest, financial or otherwise, to disclose.

## AUTHOR CONTRIBUTIONS

J.W.P., M.L., L.F.M., and H.D.P. conceived and designed research; H.H., S.T.H., L.F.M., and H.D.P. performed experiments; H.H., S.T.H., P.B., D.G.T., I.K., L.M.H., Y.X., J.W.P., R.K.K., and H.D.P. analyzed data; H.H., S.T.H., I.K., L.M.H., J.W.P., R.K.K., M.L., L.F.M., and H.D.P. interpreted results of experiments; H.H., I.K., L.M.H., and H.D.P. prepared figures; H.H. and H.D.P. drafted manuscript; H.H., S.T.H., P.B., D.G.T., I.K., L.M.H., J.W.P., R.K.K., M.L., L.F.M., and H.D.P. edited and revised manuscript; H.H., S.T.H., P.B., D.G.T., I.K., L.M.H., Y.X., J.W.P., R.K.K., M.L., L.F.M., and H.D.P. approved final version of manuscript.

## References

[1] Chalasani N, Younossi Z, Lavine JE, Charlton M, Cusi K, Rinella M, Harrison SA, Brunt EM, Sanyal AJ. The diagnosis and management of nonalcoholic fatty liver disease: practice guidance from the American Association for the Study of Liver Diseases. Hepatology 67: 328–357, 2018. doi:10.1002/hep.29367. 28714183

[2] Rinella ME, Lazarus JV, Ratziu V, Francque SM, Sanyal AJ, Kanwal F , et al. A multisociety Delphi consensus statement on new fatty liver disease nomenclature. Hepatology 78: 1966–1986, 2023. doi:10.1097/HEP.0000000000000520. 37363821 PMC10653297

[3] Younossi ZM, Koenig AB, Abdelatif D, Fazel Y, Henry L, Wymer M. Global epidemiology of nonalcoholic fatty liver disease-meta-analytic assessment of prevalence, incidence, and outcomes. Hepatology 64: 73–84, 2016. doi:10.1002/hep.28431. 26707365

[4] Liu Y, Zhong GC, Tan HY, Hao FB, Hu JJ. Nonalcoholic fatty liver disease and mortality from all causes, cardiovascular disease, and cancer: a meta-analysis. Sci Rep 9: 11124, 2019. doi:10.1038/s41598-019-47687-3. 31366982 PMC6668400

[5] Wong RJ, Singal AK. Trends in liver disease etiology among adults awaiting liver transplantation in the United States, 2014-2019. JAMA Netw Open 3: e1920294, 2020. doi:10.1001/jamanetworkopen.2019.20294. 32022875 PMC12124732

[6] Neuschwander-Tetri BA. Therapeutic Landscape for NAFLD in 2020. Gastroenterology 158: 1984–1998.e3, 2020. doi:10.1053/j.gastro.2020.01.051. 32061596

[7] Hansen HH, Feigh M, Veidal SS, Rigbolt KT, Vrang N, Fosgerau K. Mouse models of nonalcoholic steatohepatitis in preclinical drug development. Drug Discov Today 22: 1707–1718, 2017. doi:10.1016/j.drudis.2017.06.007. 28687459

[8] Zhong F, Zhou X, Xu J, Gao L. Rodent models of nonalcoholic fatty liver disease. Digestion 101: 522–535, 2020. doi:10.1159/000501851. 31600750

[9] Bergen WG, Mersmann HJ. Comparative aspects of lipid metabolism: impact on contemporary research and use of animal models. J Nutr 135: 2499–2502, 2005. doi:10.1093/jn/135.11.2499. 16251600

[10] Duvivier V, Creusot S, Broux O, Helbert A, Lesage L, Moreau K, Lesueur N, Gerard L, Lemaitre K, Provost N, Hubert EL, Baltauss T, Brzustowski A, De Preville N, Geronimi J, Adoux L, Letourneur F, Hammoutene A, Valla D, Paradis V, Delerive P. Characterization and pharmacological validation of a preclinical model of NASH in Gottingen minipigs. J Clin Exp Hepatol 12: 293–305, 2022. doi:10.1016/j.jceh.2021.09.001. 35535064 PMC9077241

[11] Pedersen HD, Galsgaard ED, Christoffersen BO, Cirera S, Holst D, Fredholm M, Latta M. NASH-inducing diets in Gottingen minipigs. J Clin Exp Hepatol 10: 211–221, 2020. doi:10.1016/j.jceh.2019.09.004. 32405177 PMC7212300

[12] Pedersen HDaM LF. Göttingen minipigs as large animal model in toxicology. In: Biomarkers in Toxicology, edited by Gupta R Elsevier, 2019, p. 75–89.

[13] Ding ZM, Xiao Y, Wu X, Zou H, Yang S, Shen Y, Xu J, Workman HC, Usborne AL, Hua H. Progression and regression of hepatic lesions in a mouse model of NASH induced by dietary intervention and its implications in pharmacotherapy. Front Pharmacol 9: 410, 2018 [Erratum in Front Pharmacol 11: 93, 2020]. doi:10.3389/fphar.2018.00410. 29765319 PMC5938379

[14] Wei G, An P, Vaid KA, Nasser I, Huang P, Tan L, Zhao S, Schuppan D, Popov YV. Comparison of murine steatohepatitis models identifies a dietary intervention with robust fibrosis, ductular reaction, and rapid progression to cirrhosis and cancer. Am J Physiol Gastrointest Liver Physiol 318: G174–G188, 2020. doi:10.1152/ajpgi.00041.2019. 31630534 PMC6985845

[15] Vacca M, Kamzolas I, Harder LM, Oakley F, Trautwein C, Hatting M , et al. An unbiased ranking of murine dietary models based on their proximity to human metabolic dysfunction-associated steatotic liver disease (MASLD). Nat Metab 6: 1178–1196, 2024. doi:10.1038/s42255-024-01043-6. 38867022 PMC11199145

[16] Schumacher-Petersen C, Christoffersen BO, Kirk RK, Ludvigsen TP, Zois NE, Pedersen HD, Vyberg M, Olsen LH. Experimental non-alcoholic steatohepatitis in Gottingen Minipigs: consequences of high fat-fructose-cholesterol diet and diabetes. J Transl Med 17: 110, 2019. doi:10.1186/s12967-019-1854-y. 30943987 PMC6448276

[17] Kleiner DE, Brunt EM, Van Natta M, Behling C, Contos MJ, Cummings OW, Ferrell LD, Liu YC, Torbenson MS, Unalp-Arida A, Yeh M, McCullough AJ, Sanyal AJ; Nonalcoholic Steatohepatitis Clinical Research Network. Design and validation of a histological scoring system for nonalcoholic fatty liver disease. Hepatology 41: 1313–1321, 2005. doi:10.1002/hep.20701. 15915461

[18] Kim D, Langmead B, Salzberg SL. HISAT: a fast spliced aligner with low memory requirements. Nat Methods 12: 357–360, 2015. doi:10.1038/nmeth.3317. 25751142 PMC4655817

[19] Anders S, Pyl PT, Huber W. HTSeq–a Python framework to work with high-throughput sequencing data. Bioinformatics 31: 166–169, 2015. doi:10.1093/bioinformatics/btu638. 25260700 PMC4287950

[20] Love MI, Huber W, Anders S. Moderated estimation of fold change and dispersion for RNA-seq data with DESeq2. Genome Biol 15: 550, 2014. doi:10.1186/s13059-014-0550-8. 25516281 PMC4302049

[21] Sergushichev AA. An algorithm for fast preranked gene set enrichment analysis using cumulative statistic calculation (Preprint). bioRxiv 060012, 2016. doi:10.1101/060012.

[22] Kanehisa M. Toward understanding the origin and evolution of cellular organisms. Protein Sci 28: 1947–1951, 2019. doi:10.1002/pro.3715. 31441146 PMC6798127

[23] Kanehisa M, Furumichi M, Sato Y, Ishiguro-Watanabe M, Tanabe M. KEGG: integrating viruses and cellular organisms. Nucleic Acids Res 49: D545–D551, 2021. doi:10.1093/nar/gkaa970. 33125081 PMC7779016

[24] Yi Y, Fang Y, Wu K, Liu Y, Zhang W. Comprehensive gene and pathway analysis of cervical cancer progression. Oncol Lett 19: 3316–3332, 2020. doi:10.3892/ol.2020.11439. 32256826 PMC7074609

[25] Napolitano F, Carrella D, Mandriani B, Pisonero-Vaquero S, Sirci F, Medina DL, Brunetti-Pierri N, di Bernardo D. gene2drug: a computational tool for pathway-based rational drug repositioning. Bioinformatics 34: 1498–1505, 2018. doi:10.1093/bioinformatics/btx800. 29236977

[26] Napolitano F, Sirci F, Carrella D, di Bernardo D. Drug-set enrichment analysis: a novel tool to investigate drug mode of action. Bioinformatics 32: 235–241, 2016. doi:10.1093/bioinformatics/btv536. 26415724 PMC4795590

[27] Govaere O, Cockell S, Tiniakos D, Queen R, Younes R, Vacca M, Alexander L, Ravaioli F, Palmer J, Petta S, Boursier J, Rosso C, Johnson K, Wonders K, Day CP, Ekstedt M, Oresic M, Darlay R, Cordell HJ, Marra F, Vidal-Puig A, Bedossa P, Schattenberg JM, Clement K, Allison M, Bugianesi E, Ratziu V, Daly AK, Anstee QM. Transcriptomic profiling across the nonalcoholic fatty liver disease spectrum reveals gene signatures for steatohepatitis and fibrosis. Sci Transl Med 12: eaba4448, 2020. doi:10.1126/scitranslmed.aba4448. 33268509

[28] Suppli MP, Rigbolt KTG, Veidal SS, Heeboll S, Eriksen PL, Demant M, Bagger JI, Nielsen JC, Oro D, Thrane SW, Lund A, Strandberg C, Konig MJ, Vilsboll T, Vrang N, Thomsen KL, Gronbaek H, Jelsing J, Hansen HH, Knop FK. Hepatic transcriptome signatures in patients with varying degrees of nonalcoholic fatty liver disease compared with healthy normal-weight individuals. Am J Physiol Gastrointest Liver Physiol 316: G462–G472, 2019. doi:10.1152/ajpgi.00358.2018. 30653341

[29] Alvarez B, Revilla C, Poderoso T, Ezquerra A, Dominguez J. Porcine macrophage markers and populations: an update. Cells 12: 2103, 2023. doi:10.3390/cells12162103. 37626913 PMC10453229

[30] Dawson HD, Lunney JK. Porcine cluster of differentiation (CD) markers 2018 update. Res Vet Sci 118: 199–246, 2018. doi:10.1016/j.rvsc.2018.02.007. 29518710

[31] Herrera-Uribe J, Wiarda JE, Sivasankaran SK, Daharsh L, Liu H, Byrne KA, Smith TPL, Lunney JK, Loving CL, Tuggle CK. Reference transcriptomes of porcine peripheral immune cells created through bulk and single-cell RNA sequencing. Front Genet 12: 689406, 2021. doi:10.3389/fgene.2021.689406. 34249103 PMC8261551

[32] Pernold CPS, Lagumdzic E, Stadler M, Mair KH, Jackel S, Schmitt MW, Ladinig A, Knecht C, Durlinger S, Kreutzmann H, Martin V, Sawyer S, Saalmuller A. Characterization of the immune system of ellegaard Gottingen minipigs - an important large animal model in experimental medicine. Front Immunol 13: 1003986, 2022. doi:10.3389/fimmu.2022.1003986. 36203585 PMC9531550

[33] Cocchetto DM, Tschanz C, Bjornsson TD. Decreased rate of creatinine production in patients with hepatic disease: implications for estimation of creatinine clearance. Ther Drug Monit 5: 161–168, 1983. doi:10.1097/00007691-198306000-00002. 6879639

[34] Liu X, Zhang H, Liang J. Blood urea nitrogen is elevated in patients with non-alcoholic fatty liver disease. Hepatogastroenterology 60: 343–345, 2013. 23858556

[35] Promrat K, Kleiner DE, Niemeier HM, Jackvony E, Kearns M, Wands JR, Fava JL, Wing RR. Randomized controlled trial testing the effects of weight loss on nonalcoholic steatohepatitis. Hepatology 51: 121–129, 2010. doi:10.1002/hep.23276. 19827166 PMC2799538

[36] Jung YK, Yim HJ. Reversal of liver cirrhosis: current evidence and expectations. Korean J Intern Med 32: 213–228, 2017. doi:10.3904/kjim.2016.268. 28171717 PMC5339475

[37] Dixon JB, Bhathal PS, Hughes NR, O'Brien PE. Nonalcoholic fatty liver disease: Improvement in liver histological analysis with weight loss. Hepatology 39: 1647–1654, 2004. doi:10.1002/hep.20251. 15185306

[38] Kuroda H, Fujiwara Y, Abe T, Nagasawa T, Oguri T, Noguchi S, Kamiyama N, Takikawa Y. Two-dimensional shear wave elastography and ultrasound-guided attenuation parameter for progressive non-alcoholic steatohepatitis. PLoS One 16: e0249493, 2021. doi:10.1371/journal.pone.0249493. 33826669 PMC8026049

[39] Takeuchi H, Sugimoto K, Oshiro H, Iwatsuka K, Kono S, Yoshimasu Y, Kasai Y, Furuichi Y, Sakamaki K, Itoi T. Liver fibrosis: noninvasive assessment using supersonic shear imaging and FIB4 index in patients with non-alcoholic fatty liver disease. J Med Ultrason (2001) 45: 243–249, 2018. doi:10.1007/s10396-017-0840-3. 29128938

[40] Joo SK, Kim W, Kim D, Kim JH, Oh S, Lee KL, Chang MS, Jung YJ, So YH, Lee MS, Bae JM, Kim BG. Steatosis severity affects the diagnostic performances of noninvasive fibrosis tests in nonalcoholic fatty liver disease. Liver Int 38: 331–341, 2018. doi:10.1111/liv.13549. 28796410

[41] Huang Z, Zhou J, Lu X, Zhang T, Xu S, Jin J, Zheng R, Chen S. How does liver steatosis affect diagnostic performance of 2D-SWE.SSI: assessment from aspects of steatosis degree and pathological types. Eur Radiol 31: 3207–3215, 2020., doi:10.1007/s00330-020-07288-5. 33119813

[42] Arena U, Vizzutti F, Abraldes JG, Corti G, Stasi C, Moscarella S, Milani S, Lorefice E, Petrarca A, Romanelli RG, Laffi G, Bosch J, Marra F, Pinzani M. Reliability of transient elastography for the diagnosis of advanced fibrosis in chronic hepatitis C. Gut 57: 1288–1293, 2008. doi:10.1136/gut.2008.149708. 18448567

[43] Fraquelli M, Rigamonti C, Casazza G, Donato MF, Ronchi G, Conte D, Rumi M, Lampertico P, Colombo M. Etiology-related determinants of liver stiffness values in chronic viral hepatitis B or C. J Hepatol 54: 621–628, 2011. doi:10.1016/j.jhep.2010.07.017. 21146243

[44] Kang BK, Lee SS, Cheong H, Hong SM, Jang K, Lee MG. Shear wave elastography for assessment of steatohepatitis and hepatic fibrosis in rat models of non-alcoholic fatty liver disease. Ultrasound Med Biol 41: 3205–3215, 2015. doi:10.1016/j.ultrasmedbio.2015.07.025. 26349582

[45] Nalbantoglu IL, Brunt EM. Role of liver biopsy in nonalcoholic fatty liver disease. World J Gastroenterol 20: 9026–9037, 2014. doi:10.3748/wjg.v20.i27.9026. 25083076 PMC4112884

[46] Engel RW. Anemia and edema of chronic choline deficiency in the rat. J Nutr 36: 739–749, 1948. doi:10.1093/jn/36.6.739. 18100938

[47] Johnson BC, James MF. Choline deficiency in the baby pig. J Nutr 36: 339–349, 1948. doi:10.1093/jn/36.3.339. 18881998

[48] Schaefer AE, Copeland DH, Salmon WD. Duodenal ulcers, liver damage, anemia and endema of chronic choline deficiency in dogs. J Nutr 43: 201–221, 1951. doi:10.1093/jn/43.2.201. 14851039

[49] Li Y, Liu L, Wang B, Wang J, Chen D. Hematocrit is associated with fibrosis in patients with nonalcoholic steatohepatitis. Eur J Gastroenterol Hepatol 26: 332–338, 2014. doi:10.1097/MEG.0000000000000015. 24172912

